# The Association of Smoking and Coffee Consumption With Occurrence of Upper Gastrointestinal Symptoms in Patients With Active Helicobacter pylori Infection in Jazan City: A Cross-Sectional Study

**DOI:** 10.7759/cureus.33574

**Published:** 2023-01-09

**Authors:** Alfadl A Abdulfattah, Hanan A Jawkhab, Altaf A Alhazmi, Nada A Alfaifi, Maryam A Sultan, Rajaa A Alnami, Nada Y Kenani, Shorooq A Hamzi, Shahd M Abu Sharha, Ibrahim M Dighriri

**Affiliations:** 1 Department of Internal Medicine, Jazan University, Jazan, SAU; 2 Faculty of Medicine, Jazan University, Jazan, SAU; 3 Facility of Medicine, Jazan University, Jazan, SAU; 4 Faculty of Medicine, Jazan University, Jazan, SAU; 5 Department of Pharmacy, King Abdulaziz Specialist Hospital, Taif, SAU

**Keywords:** jazan, h pylori, helicobacter pylori infection, gastrointestinal, coffee, smoking

## Abstract

Background: *Helicobacter pylori* (*H. pylori*) is a severe infection responsible for upper gastrointestinal symptoms (UGISs). Several causes of *H. pylori* infection include food ingestion and person-to-person transmission. Many lifestyle variables can affect the occurrence of UGISs such as coffee consumption and smoking.

Objective: To assess the association between smoking and coffee consumption and the occurrence of UGISs in patients with active H. pylori infection in Jazan city in Saudi Arabia.

Methodology: A descriptive cross-sectional research design was used to conduct the study between July 2022 and August 2022 in Jazan, southern Saudi Arabia. Male and female Saudis or non-Saudis ≥ 18 years of age with an active *H. pylori* infection were included. Participants under 18 years or without active *H. pylori* infection were excluded. Data were collected from participants using the convenience sampling technique and a structured questionnaire. The first part of the questionnaire evaluated social and demographic factors such as age, sex, place of residence, nationality, and educational level; the second part evaluated smoking and coffee-drinking habits. Furthermore, frequencies and percentages represented categorical variables. A continuous variable was converted to a categorical variable. The relationship between different variables is tested using the Chi-square test.

Result: The total number of respondents who completed the questionnaire was 1225, with only 422 having *H. pylori* entries in this study. There were 290 (68.7%) men and only 132 (31.3%) women among them; the majority were young adults (18-20 years old). More than half of the participants (53%) never smoked, 23% were active smokers, and 23% were former smokers. Around 27.1% smoke five cigarettes a day and 12.6% smoke five to 15 cigarettes a day. Three-hundred (71.1%) of the participants drank coffee. 23.9% indicated that they did not drink coffee. Of those who take coffee, more than half (51.7%) take fewer than three cups daily and 25.6% take approximately three to five cups of coffee per day. Our findings indicate a link between coffee consumption and UGISs (p = 0.00), while smoking did not have a significant relationship with UGISs (p = 0.06).

Conclusion: Our research showed that drinking coffee was related to UGISs, but smoking was not found in people with active *H. pylori* infection. In smokers, UGISs increased substantially, but not significantly. We need real-world research to identify the association between coffee consumption and UGISs. In addition, we need to educate people at risk for UGISs to reduce coffee, smoking, and other risk factors.

## Introduction

*Helicobacter pylori* (*H. pylori*) is a spiral-shaped gram-negative bacillus that was first identified in 1982 [1]. The World Health Organisation (WHO) has identified *H. pylori* as the first bacteria recognized to cause cancer. Also, it causes chronic gastritis, peptic ulcers, and gastric adenocarcinoma [2]. The prevalence of *H. pylori* infection is believed to be 50% worldwide, with developing countries having the highest disease rates [3]. *H. pylori* in the stomach cause gastric cancer and peptic ulcer, resulting in more than a million people dying annually [4,5]. Most people with *H. Pylori* infection don't have any symptoms or problems. However, bacteria can damage the inner protective lining and cause gastritis or peptic ulcer symptoms, such as dull or searing stomachache, sudden weight loss, bloating, nausea and diarrhea, indigestion, burping, decreased appetite, and black stools [6].

The most common ways *H. pylori* spreads are through eating contaminated food and from person to person through oral and saliva [2]. Age, sex, and blood type factors increase the risk of *H. pylori* infection. At the same time, the association between *H. pylori* infection and lifestyle factors is vague. However, smoking has been associated in multiple studies with the onset of H. pylori infection, its increased persistence, and the lower efficacy of its eradication. This may increase smokers' risk of stomach cancer, although this has not been consistently demonstrated. Active H pylori infection with smoking may have somewhat antagonistic effects on increased acid, pepsin, and stomach motility [7]. Also, coffee consumption has been associated with peptic ulcer disease and gastro-oesophageal reflux disease. Coffee may cause a variety of disorders because it stimulates stomach acid secretion [8]. *H. pylori* infection can be diagnosed in noninvasive ways such as serology, urea breath testing, and fecal antigen testing. Histopathological examination, rapid urease test, and polymerase chain reaction are some invasive diagnostics performed during endoscopy [9]. A combination treatment regimen can treat *H. pylori* infections, and standard triple therapy includes antibiotics and a proton pump inhibitor [10]. This study aims to assess the relationship between smoking, coffee consumption, and upper gastrointestinal symptoms (UGISs) in patients with active *H. pylori* infection.

## Materials and methods

Design and period of study

Descriptive cross-sectional research was conducted using a self-administered anonymous questionnaire. Between July 2022 and August 2022, the questionnaire was distributed along with a statement about the proposed study and a consent form. The questionnaire was distributed online to patients in general medical clinics in Jazan City, southern Saudi Arabia.

Study sample

Raosoft's sample size calculator (http://www.raosoft.com/samplesize.html) was used to calculate the sample size. The required sample size was 400. Data were acquired using a convenient sampling method.

Inclusion criteria

Male and female Saudis or non-Saudis living in Jazan city having an age ≥ 18 years with active *H. pylori *infection were included in this study.

Exclusion criteria

Participants less than 18 years or those not having an active *H. pylori *infection, people from outside Jazan city or who refused to participate in the study or did not complete the questionnaire were excluded from the study.

Data collection

Google Sheets was used to design the standard electronic questionnaire in Arabic. The first part of the questionnaire asked about social and demographic factors such as age, sex, place of residence, nationality, and education level; the second part asked about smoking and drinking coffee.

Pilot study

The feasibility, objectivity, clarity, and applicability of the proposed solution were examined in 15% of the pilot study. Furthermore, the research sample was modified and retested. The results of the pilot study were not included in the results section.

Ethical consideration

After receiving approval from Jazan University's Standing Committee for Scientific Research reference number: REC-44/02/309, we began data collection. Data collection was carried out with the utmost care for the participants' privacy. In addition, participants were asked for their voluntary online consent before the study began.

Statistical test

The SPSS program Version 28.0 (IBM Corp., Armonk, NY) was used to analyze the data obtained. After that, the information was sorted and fed into SPSS. We converted a continuous variable, such as age, to a categorical variable. Frequencies and percentages represented categorical variables. We evaluated the relationship between different variables using the Chi-square test to determine whether they were significant. We then considered the data important if the P-value was less than 0.05.

## Results

**Figure 1:**
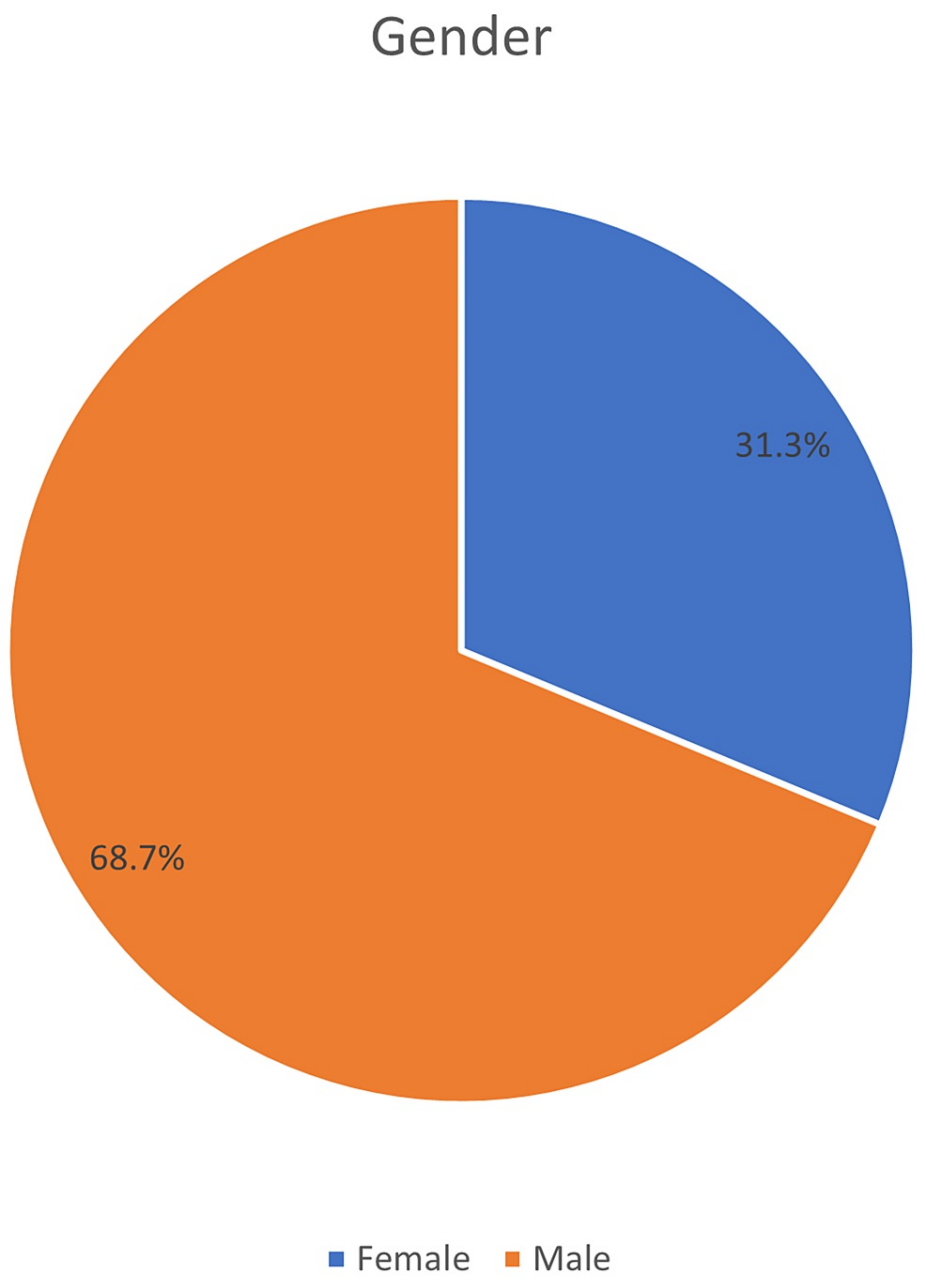
Gender distribution of the respondents.

The total number of respondents who completed the questionnaire was 1225, with only 422 having *H. pylori* entries in this study. The majority of respondents 290 (68%) were male, and only 132 (31.3%) were women (Figure 1).

The majority, the youngest, constituted between 18 and 20 years (64%). This was followed by ages 21-30 (23%), then 41-50 (7.6%) years. The minority was the elderly, 23 (5.5%) of the entire 422 population of the study. One-hundred and forty-one (33.4%) of the respondents have obtained college degrees, 29.9% had a high school education, 28.9% had a university education, and 6.6% had a lower level of education than high school.

More than half of the participants in this study had never smoked (53%). About 23% of the population consisted of active and former smokers. Most were current and former smokers, 38% smoked cigarettes or cigars, while only 1.2% used chewing tobacco. About 27% of these current or former smokers had a smoking frequency of five cigarettes per day. Twelve percent of current or former smokers had a frequency between five and 15 times per day. Other types of smoking were negligibly rare among participants in this investigation. Three hundred (71.1%) of the participants drank coffee, 23.9% indicated that they did not take coffee, while 5% stated that they had taken coffee. Of those who take coffee, more than half (51.7%) take less than three cups daily and 25.6% take approximately three to five cups of coffee per day. About 22% of the respondents who take coffee consume more than five cups (Table 1).

**Table 1:** Sociodemographic characteristics of 422 respondents

Sociodemographic characteristic		Frequency	Percent
Age	18-20	270	64.0
	21-30	97	23.0
	41-50	32	7.6
	51 or above	23	5.5
Education	Less than high school	28	6.6
	High school	126	29.9
	College	141	33.4
	University degree	122	28.9
	None	5	1.2
Marital status	Single	217	51.4
	Married	185	43.8
	Divorced	15	3.6
	Widow	5	1.2
Nationality	Saudi	411	97.4
	Non-Saudi	11	2.6
Residence	Urban	323	76.5
	Rural	99	23.5
Smoking	Never smoked	225	53.3
	Past smoker	99	23.5
	Current smoker	98	23.2
Smoking methods	Cigarettes or cigars	161	38.2
	Chewing tobacco	5	1.2
	Hookah	49	11.6
Number of smoking/cigarettes per day	< 5/day	114	27.1
	5-15/day	53	12.6
	>15/ day	36	8.6
Do you drink Coffee currently	Yes	300	71.1
	No	101	23.9
	Past drinker	21	5.0
Coffee consumption (cups/day)	Less than 3	218	51.7
	From 3 to 5	108	25.6
	More than 5	96	22.7

The findings indicate a link between coffee consumption and the occurrence of UGISs (p = 0.00), indicating that the results are statistically significant. Smoking, on the other hand, did not show a relationship (p = 0.06) (Table 2).

**Table 2:** The association between smoking and coffee consumption and the occurrence of UGISs in patients with active H. pylori infection.

Variables		Occurrence of UGISs			P-Value
		Yes	No	Total	
Smoking	Never smoked	130	95	225	0.067
	Past smoker	68	31	99	
	Current smoker	52	46	98	
Coffee consumption	Yes	196	104	300	0.000
	No	36	65	101	
	Past drinker	18	3	21	

## Discussion

Our findings indicate a link between coffee consumption and UGISs, while smoking did not link to UGISs. There was a substantial, but not considerable, increase in the occurrence of UGISs among smokers. Past studies revealed that UGISs had a negative dose-response relationship with smoking and a positive relationship with coffee [11].

Our findings on how smoking, UGISs, and *H. pylori* infection are related are consistent with previous research [12]. Despite variations in study populations and methodologies, most studies revealed that smokers had a slightly higher risk of *H. pylori* infection [11,12]; however, these odds were only statistically significant in two studies [13,14]. Due to the weakness of most single observational studies, these patterns are consistent with a probable minor increase in active *H. pylori *infection resulting from smoking.

Findings indicating a significant association between smoking and active *H. pylori *infection may result from many mechanisms with partly inhibitory action on infection risk [15]. The release of pepsin and acid can be increased, and smoking can affect digestive motility, prostaglandin synthesis, gastric mucosal blood flow, and mucus production [15].

Microbial and environmental variables interact to affect the health of the stomach. Everyday interactions between the immune system and the living microorganisms keep stomach inflammation under control [16,17]. Some beneficial bacteria, such as prebiotics and probiotics, are consumed with traditional food products and produce antimicrobials and antioxidants, which are essential for maintaining the homeostasis of the gastric environment. To keep an individual healthy stomach, significantly when pH is decreased, *Actinobacteria* and *Firmicutes* are the only microbial phyla that can colonize the stomach [16,17]. *H. pylori* colonization also causes the loss of stomach parietal cells and an elevation in gastric pH, in addition to damaging the stomach owing to virulence aspects, such as vacuolating cytotoxin. Numerous dangerous microorganisms could dominate in altered gastrointestinal pH. Other factors may also have the ability to disrupt stomach homeostasis. For instance, increasing red meat consumption, increasing nonsteroidal anti-inflammatory drugs, and excessive use of acid suppressors can all exacerbate the pro-inflammatory response. Intestinal microbiota dysbiosis, especially the reduction of *Bifidobacterium* species, has recently been related to severe stomach diseases caused by *H. pylori* [16,17].

The findings of a cohort study among epidemiologists, which found a 4.6-fold increase in the risk of variation from adverse to positive outcomes for *H. pylori* in serum, are consistent with the positive relationship between coffee consumption and *H. pylori* infection found in our study [18]. In the epidemiological investigation, seroconversion was defined as the change from negative to positive outcomes for antibodies to *H. pylori* in serum [19].

Our study has a few limitations, as the cross-sectional design and convenience sampling used cannot confirm the causal association between the compared variables. Self-reported responses may overestimate or underestimate the results. In addition, the subjects for the study were chosen from a particular region, and thus they may not have been the representatives of the entire country. Considering that patients were included through social media, there could be a risk of selection bias. The strength of this study was a representative mix of subjects from all different age groups and socioeconomic classes. We recommend that a larger nationwide study involving different regions be conducted to learn about the trends of increasing UGISs in patients with active *H. pylori* infection in the general population.

## Conclusions

The infection caused by *H. pylori* is contagious and a threat to public health because it can affect people of any age. According to the results of our research, drinking coffee was related to UGISs, while smoking did not link UGISs in respondents who had active *H. pylori *infection. There was a substantial, but not considerable, increase in the occurrence of UGISs among smokers. We need to do actual-world research to identify the relationship precisely. Also, we need to educate patients with a risk of developing UGISs, to decrease coffee consumption, smoking, and other factors that may increase complications of UGISs.

## References

[1] Gravina AG, Priadko K, Ciamarra P (2020). Extra-gastric manifestations of Helicobacter pylori infection. J Clin Med.

[2] Alexander SM, Retnakumar RJ, Chouhan D (2021). Helicobacter pylori in human stomach: the inconsistencies in clinical outcomes and the probable causes. Front Microbiol.

[3] Dhakal OP, Dhakal M (2018). Prevalence of Helicobacter pylori infection & pattern of gastrointestinal involvement in patients undergoing upper gastrointestinal endoscopy in Sikkim. Indian J Med Res.

[4] (2017). Global, regional, and national age-sex specific mortality for 264 causes of death, 1980-2016: a systematic analysis for the Global Burden of Disease Study 2016. Lancet.

[5] Bray F, Ferlay J, Soerjomataram I, Siegel RL, Torre LA, Jemal A (2018). Global cancer statistics 2018: GLOBOCAN estimates of incidence and mortality worldwide for 36 cancers in 185 countries. CA Cancer J Clin.

[6] Spee LA, Madderom MB, Pijpers M, van Leeuwen Y, Berger MY (2010). Association between helicobacter pylori and gastrointestinal symptoms in children. Pediatrics.

[7] Ferro A, Morais S, Pelucchi C (2019). Smoking and Helicobacter pylori infection: an individual participant pooled analysis (Stomach Cancer Pooling- StoP Project). Eur J Cancer Prev.

[8] Boekema PJ, Samsom M, van Berge Henegouwen GP, Smout AJ (1999). Coffee and gastrointestinal function: facts and fiction. A review. Scand J Gastroenterol Suppl.

[9] Agarwal PK, Badkur M, Agarwal R, Patel S (2018). Prevalence of Helicobacter pylori infection in upper gastrointestinal tract disorders (dyspepsia) patients visiting outpatient department of a hospital of North India. J Family Med Prim Care.

[10] Hu Y, Zhu Y, Lu NH (2020). Recent progress in Helicobacter pylori treatment. Chin Med J (Engl).

[11] Moayyedi P, Forman D, Braunholtz D, Feltbower R, Crocombe W, Liptrott M, Axon A (2000). The proportion of upper gastrointestinal symptoms in the community associated with helicobacter pylori, lifestyle factors, and nonsteroidal anti-inflammatory drugs. Am J Gastroenterol.

[12] Nordenstedt H, Graham DY, Kramer JR (2013). Helicobacter pylori-negative gastritis: prevalence and risk factors. Am J Gastroenterol.

[13] Woodward M, Morrison C, McColl K (2000). An investigation into factors associated with Helicobacter pylori infection. J Clin Epidemiol.

[14] Parsonnet J, Friedman GD, Orentreich N, Vogelman H (1997). Risk for gastric cancer in people with CagA positive or CagA negative Helicobacter pylori infection. Gut.

[15] Birnie DH, Holme ER, McKay IC, Hood S, McColl KE, Hillis WS (1998). Association between antibodies to heat shock protein 65 and coronary atherosclerosis. Possible mechanism of action of Helicobacter pylori and other bacterial infections in increasing cardiovascular risk. Eur Heart J.

[16] Devi TB, Devadas K, George M (2021). Low Bifidobacterium abundance in the lower gut microbiota is associated with Helicobacter pylori-related gastric ulcer and gastric cancer. Front Microbiol.

[17] Gao JJ, Zhang Y, Gerhard M (2018). Association between gut microbiota and Helicobacter pylori-related gastric lesions in a high-risk population of gastric cancer. Front Cell Infect Microbiol.

[18] Chang WL, Yeh YC, Sheu BS (2018). The impacts of H. pylori virulence factors on the development of gastroduodenal diseases. J Biomed Sci.

[19] Veldhuyzen van Zanten SJ, Pollak PT, Best LM, Bezanson GS, Marrie T (1994). Increasing prevalence of Helicobacter pylori infection with age: continuous risk of infection in adults rather than cohort effect. J Infect Dis.

